# The Transformation of Conflicts into Relational Well-Being in Physical Education: GIAM Model

**DOI:** 10.3390/ijerph18031071

**Published:** 2021-01-26

**Authors:** Aaron Rillo-Albert, Pere Lavega-Burgués, Queralt Prat, Antoni Costes, Verónica Muñoz-Arroyave, Unai Sáez de Ocáriz

**Affiliations:** 1 Motor Action Research Group (GIAM), INDEST, Institut Nacional d’Educació Física de Catalunya (INEFC), Universitat de Barcelona (UB), 08007 Barcelona, Spain; usaez@gencat.cat; 2 Motor Action Research Group (GIAM), INDEST, Institut Nacional d’Educació Física de Catalunya (INEFC), Universitat de Lleida (UdL), 25003 Lleida, Spain; plavega@inefc.es (P.L.-B.); qprat@gencat.cat (Q.P.); tcostes@inefc.udl.cat (A.C.); veronicarroyave15@yahoo.es (V.M.-A.)

**Keywords:** motor praxeology, coexistence, pedagogical model, traditional sporting games, education of motor conducts

## Abstract

To educate the relational well-being in order to learn to live together in society is one of the main needs of modern physical education (PE). Teachers are in need of pedagogical models to instruct peaceful coexistence and transform possible conflicts into PE. The goal of this study was to determine the effect of a pedagogical model (GIAM model) designed for conflict transformation on the relational well-being of students in obligatory secondary school (ESO in Spain). This study was an empirical research (associative strategy, comparative study using mixed methods). A number of 287 valid students (*M* = 14.90; *SD* = 0.66) participated in this study from 4 different secondary schools (SSs): third ESO (SS_1_ (*n* = 75); SS_3_ (*n* = 45); SS_4_ (*n* = 86)) and fourth ESO (SS_2_ (*n* = 81)). A sequence of seven learning sessions was conducted, the intervention of the teachers on the GIAM model and the student’s motivational climate perception caused by this learning sequence was analyzed. The teachers who best adapted their intervention to the GIAM model obtained greater significant changes (*p* < 0.005) in favor of the relational well-being of their students. This research provides scientific evidence and intervention strategies for students to learn how to transform the conflicts, adopting a collaborating style based on reflection-for, -in- and on-motor action.

## 1. Introduction

Different international reports on education indicate the importance of school coexistence to create high-quality learning experiences based on democratic coexistence [1].

In this context, schools operate as small communities, formed by students with increasingly more diverse characteristics, and as a result of the continuous social changes [2]. In obligatory secondary school (ESO in Spain), students face the fact that they must learn to be in contact with their peers, being immersed themselves in maturing and building individual and group identity [3]. For this reason, at this stage of education one of the main educational challenges is learning to live together proactively [4].

Interpersonal relationships with other educational agents (e.g., school: students, teachers; family: parents; etc.) often result in tensions that lead to conflicts. A lack of managing them constructively could affect their relational well-being and the positive climate of the class group. Depending on the seriousness of the conflicts, very negative consequences can arise within and outside the school context [5,6]. Very negative consequences could arise inside and outside the school context, depending on the seriousness of the conflicts.

However, a conflict should not always be seen exclusively as a negative experience [7], as they also represent opportunities to learn how to transform interpersonal tensions and lead them to relational well-being [8,9,10,11,12]. Learning to interact involves making respectful decisions with others (relational intelligence) and knowing how to enjoy meeting others (socio-emotional intelligence). All these factors can have a positive impact on students’ everyday life and on their future working and social life [9].

### 1.1. Relational Well-Being and Motivational Climate

Generating learning contexts, associated with student’s positive motivational climate, is one of the key aspects for the improvement of interpersonal relationship and relational well-being [13,14].

According to the achievement goal theory (AGT), the motivational climate is the set of signs that indicate satisfaction or frustration of the participants in a certain context of intervention according to whether or not their basic psychological needs are met: autonomy, competence, and relatedness [15,16,17,18].

This theory holds that the motivational climate may have two orientations: (a) towards the task, when progress, equality, relationship, and cooperation between partners are important aspects in goal achieving, and (b) towards the ego, when the achievement of goals becomes the main goal, where social comparison may generate environments of rivalry [18,19].

In a complementary way, the self-determination theory (SDT) explains that motivation comes from the actions carried out in the different activities proposed. Emphasizing different types of motivations: (a) intrinsic motivation, where the internal aspects of the activity are related to well-being and progress in task-oriented skills with processes of pleasure and participation; (b) the extrinsic motivation, derived from external aspects, which prioritizes social recognition; and (c) demotivation, resulting from a lack of intrinsic and extrinsic motivation [20,21,22,23].

In this sense, as both theories confirm (AGT and SDT), relational well-being shall be benefited if the orientation to task-climate prevails over the orientation to ego-climate, where conflicting situations may arise by putting individual goals before team ones [18,19,22,24].

### 1.2. Relational Well-Being and Education of Motor Conducts

Physical education (PE) is a subject that offers an ideal scenario to teach interpersonal relationships using procedural learning.

The PE teacher has a complete set of motor situations, either with games, sports, or other activities. Each motor situation tests the students who will try to overcome it, keeping motor and relational perspectives in mind as other classmates join them frequently.

The theory of motor action or motor praxeology [25] explains that any game has an internal logic (IL) or identity card that tests students on solving problems associated with a unique way of interacting with others (e.g., cooperative relationships with allies, opposition relationships with rival, etc.); interacting with physical space (e.g., zone occupation); interacting with time (e.g., perform according to a specific moment in the game, for example, the scoreboard), and interacting with equipment (e.g., how to use the ball in basketball). Each set has permanent structural features also named an IL, regardless of the participants’ qualities.

However, each student reacts differently confronting the same experience. To understand the meaning and significance of students’ motor responses to any motor situation, it is necessary to change the classical visions that only consider the motor execution and the performance of motor actions. To this end, the science of motor action provides the concept of motor conduct (MC). This concept departs from the traditional use of other terms such as the movement, skills, or abilities focused primarily upon the decontextualized motor execution of the motor task [25]. The MC implies that the motor execution performed by the student, (external observation or motor behavior) and the internal meaning that has this intervention (internal observation) are seen in a unitary way. To this end, it is necessary to understand that any motor action in a game (e.g., Ricard pass the ball to Irene in a basketball game) involves an organic execution (a pass made with a certain strength, speed or precision). However, at the same time, the pass implies a decision-making (cognitive dimension) having decided to pass instead of shooting; the pass also has a social significance (relational dimension), having passed the ball to Irene and not to Ashley; the same pass also includes an affective meaning (emotional dimension) of joyfully showing the outcome of that action. Thus, any student who takes part in a game is the leading role of unique motor actions, that is to say, meaningful MCs which bear witness to how the educator takes part in each motor situation [26]. This is a profound experience in which all dimensions of the person are activated in an interlocking way [27].

Depending on the pursued or expected educational effects [25], PE teacher interprets the students’ MCs when they try to adapt to the IL of the game in which they participate. The teacher needs to identify the different groups of MCs according to these pedagogical effects. In a programme that aims to educate the relational well-being (interpersonal relationships), MCs can be classified into three groups depending on whether or not they adjust to the game’s IL [28]: (a) adjusted motor conduct (AMC), related to responses that meet the relational requirement of the IL (e.g., pass the ball effectively to a partner; to oppose an opponent with respect when she/he tries to beat us with the ball, etc.); (b) misadjusted motor conduct (MMC), associated with responses that differ from the relational requirements established by the IL (e.g., to pass the ball too hard intentionally to prevent a teammate from receiving it correctly, etc.), and (c) perverse motor conduct (PMC), referred to responses that are not allowed by the rules of the game (e.g., touching the ball with your feet in basketball; push an opponent, etc.).

Different types of verbal conducts (VCs) can also be identified in team games when players agree on team strategy (VCAS). These conducts before the game can also be adjusted, misadjusted, or perverse depending on whether they favor or disfavor that group agreement [26,29].

When a student’s MCs are not directed towards relational well-being with other players (VCs or MCs misadjusted or perverse) generate tension in interpersonal relationships and cause negative emotions. If this tension is not managed constructively, it can trigger conflicts [10].

To intervene in conflict transformation, it must be characterized and understood as an interactive process. In this study, we use the concept of motor conflict [29]. From this point of view, any conflict is made up of two parts: the origin that arises from an «action» originated from a conduct VCAS, MMC, and PMC (misadjusted or perverse) which leads to a «reaction» (response) from the other party, with verbal aggression (e.g., insult), physical aggression (e.g., push), or a combination (both verbal and physical) [30].

Once a conflict is identified, the next step is to know the intensity of the relational tension. Based on the criteria described, the teacher can use the conflict index (IC*f*). The IC*f* is the sum of the intensity level of the generating agent (1-VCAS; 2-MMC; 3-PMC) and the intensity of the conflicting response to the previous stimulus (1-Verbal Aggression; 2-Physical Aggression; 3-Mixed Aggression). The least intense conflicts have a rating of 2 and the most intense 6 [31].

Once the parties of the conflict and the level of intensity have been identified, the time comes for the teacher to act. The PE conceived as education of MCs means to transform conflicting conducts (verbal and/or misadjusted or perverse motor conducts) into VCs and MCs adjusted [31]. Two main areas of action for teachers have been identified [25,29,30].

(a) Intervention on the game (IL), for example, changing the rules (e.g., resizing the playing field, modifying the number of players, changing the way to win a point, etc.). These teacher’s actions mainly influence the beginning of the conflict regarding the conditions of the game.
(b) Intervention on the attitude of the protagonists towards the conflict, encouraging self-management and constructive reflection. These active interventions performed by the teacher mainly affect the reaction to conflicting conduct. In this section, it is necessary to understand how the subjects have dealt with the conflict, that is to say, the attitude adopted. Among the different theoretical approaches, as an example, we mention the contribution of the Dual Concern Model [32] and later interpretations [33,34]. This model classifies five styles or attitude modes in conflict management, depending on whether the emotional orientation is directed towards assertiveness (to satisfy one’s concerns) and/or the cooperativeness (meeting the concerns of others). Table 1 shows five attitudes in terms of ways of dealing with conflict, the importance of both dimensions and winning the game [35,36].

The challenge for the teacher is to educate interpersonal relationships, transforming relational tensions into a style or attitude mode towards win–win conflict (in this conflict context, referred as collaborating) [36,37]. Both sides of the conflict should find a way to move towards positive attitudes of self-esteem, empathy, active listening, or consensual decision-making [9,38,39]. From this perspective, the adoption of the Attitudinal Styles in PE [40,41] can be of great interest in transforming conflicts.

This style is close to approaches in which there is a high level of participation, especially in the form of student cooperation [40]. This approach should be integrated into any pedagogical model for conflict education. The Attitudinal Style in PE integrates: (a) the selection of the most appropriate activities according to the set goals; (b) the sequential organization of the students’ intervention, accompanied by a reflective process into attitudes towards the conflict; and (c) the final assembly or intervention in which the students can be encouraged to cooperate towards a common goal, valuing their contribution and respect for others. All this will contribute to the improvement of self-esteem (subjective well-being) and coexistence (relational well-being) [8,39,41].

The transformation of attitude as well as supporting the subjective and relational well-being of the students it also implies an affective transformation, as we move from states of socio-emotional discomfort (negative emotions) to states of socio-emotional well-being (positive emotions) [10,42].

It is essential that the student discovers the meaning of the activities, understands the goals to be achieved and the interest for her/his training. In addition, she/he must recognize how her/his intervention has gone both in the adaptation to the rules of the game, and how she/he interacted with other participants. Some studies performed using this approach methodology confirmed that positive transformation of conflicts is possible with students of different age [30,31,43].

In the event of conflicts, it is convenient to identify the circumstances in which they have arisen and to recognize the style or attitude mode adopted. Thus, little by little, progress can be made in adopting commitments for future interventions [44,45,46,47].

The aim is to encourage students in meaningful learning, hence, building awareness among individual and collective reflection will be a key teaching strategy in this training process.

The reflection as a cognitive and affective process or activity that (1) requires active engagement on the part of the individual; (2) is triggered by an unusual or perplexing situation or experience; (3) involves examining one’s responses, beliefs, and premises in light of the situation at hand; and (4) results in the integration of the new understanding into one’s experience [48] (p. 41).

This reflection process can be promoted through different moments or circumstances of the session: (a) reflection-for-action, to favor the positive group agreement on some team strategy before starting a game or to reduce problems related to the game; (b) reflection-in-action, at the time of identifying a conflict; (c) reflection-on-action, in the aftermath of the conflict, in the same session or at the beginning of the next session [44,49,50,51,52,53].

With all this, the transformation of meaningful interpersonal relationships will allow the student to direct her/his attention to the task and the group, rather than towards selfish interests. Thus, the intrinsic motivation of the students will be promoted, that is to say, the motivational orientation towards the task-climate [18,40,54].

### 1.3. GIAM Model: Transforming Conflictive Motor Conducts through Physical Education

Based on the previous theoretical considerations, this article describes the GIAM pedagogical model which consists of different phases and actions to intervene in a PE session or learning sequence (unit made up of several sessions), in order to transform conflicts in a positive way.

This is a pedagogical model [55] as it identifies the learning outcomes to be achieved (to transform conflicts) and teaching strategies (based on reflection-for, -in- and on-motor action, constructive and cooperative dialogue, modification of game situations, etc.). The GIAM model could be used in any PE program and also in sports initiation.

The GIAM model phases are described below. This model is structured around a series of preparatory actions (phase 1 and 2) and a series of actions to be carried out during the session or learning sequence (phase 3).

**Figure 1:**
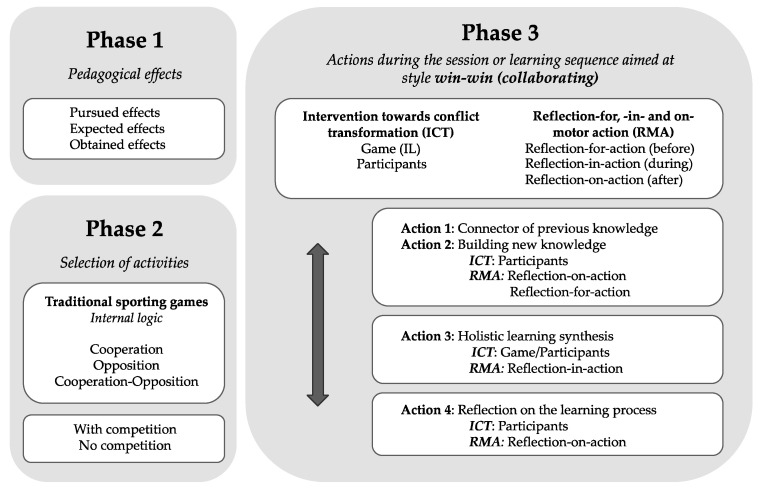
Diagram of the GIAM model.

The teacher uses the initial phases to determine the pedagogical effects of one’s learning sequence. According to these effects the teacher should select the activities that allow to test the students (appropriate IL). During the games, the teacher has a series of didactic strategies aimed at conflict transformation, to intervene in the game or in the attitude of the participants through reflection-for, -in- and on-motor action based on constructive and cooperative dialogue (see Figure 1).

#### 1.3.1. Phase 1: Pedagogical Effects

Before starting to design an educational intervention or project is necessarily required to establish the pursued or expected pedagogical effects (educational competencies to be accredited by the student [56]). This first step will allow making an objective comparison between the pursued effects and the effects obtained (learning outcome achieved after the pedagogical intervention) [25].

MCs education involves recognizing that students’ learning must consider their strictly motor involvement (e.g., the technical ability to pass or receive a ball) but without forgetting about the organic involvement (energy management), cognitive (decision-making), social (relationship with others), and emotional (use and management of emotions). In the case of education for relational well-being and conflict, the focus is on the pursued or expected effects in the socio-emotional field.

The expected effects should show whether the pupils have improved their motor skills after their intervention in the games through MCs. Being competent when taking part in a game means to have integrated different knowledge in a unitary way: (a) the student should know how to interpret the IL of the game in which she/he is going to intervene, figuring out the main social problems to be solved, linked to the relationship with the other participants (allies and/or rivals), material, space, and time (learning to know and cognitive orientation); (b) all that declarative knowledge, must be put into action in any game situation, accepting the rules of the game and making respectful interpersonal relationships that favor a positive climate of relational well-being (learning to live together and the relational dimension). In addition, (c) this procedural learning, will also be associated with the regulation of negative emotions in situations of tense relationships or conflicts with others and the desire to improve (learning to be and emotional orientation) [57] (see Figure 1).

#### 1.3.2. Phase 2: Activity Selection

PE has a wide range of resources (games, sports, expressive activities, etc.). Among this broad range of activities, traditional sporting games (TSG) deserve special attention [58]. These are games with original rules, linked to local tradition or culture. They trigger a great diversity of motor relationships [59,60,61]. According to the theory of motor action or motor praxeology [25], depending on the type of motor relationship, four domains of motor action can be distinguished: (a) psychomotor games, absence of motor interaction (e.g., jumping rope); and (b) sociomotor games, presence of motor interaction between participants, classified in: (a) cooperative games, where two or more participants interact constructively towards a common goal (e.g., construction of a human tower); (b) opposition games, where several participants oppose each other as to the achievement of the goal (e.g., fighting games, chasing “it” games); and (c) cooperation-opposition games, where a team of participants work together while opposing another team (opposing team) to reach the goals (e.g., dodgeball).

In parallel, these four families of games can be played with the presence or absence of competition (final score). As there is a scoreboard, at the end of the game, winners and losers are distinguished and this brings extra pressure. When there is no score, games do not have a clear ending and do not compare player results (e.g., dancing games, chasing games changing rivals, games changing teams during the game, etc.).

There is scientific evidence that games with different characteristics (a type of motor interaction or competition) trigger unequal effects on students [42,62,63]. For this reason, there must be a clear correspondence between the expected effects and the type of games or activities to be selected.

If an educational project aims to educate interpersonal relationships, sociomotor games (collective) are the most appropriate, as their IL requires them to enter into a relationship with others. Among these activities, games with opponents and competition are those that can cause misadjusted or perverse MCs associated with tensions (negative emotions) in interactions with others [57]. These conducts must be transformed as they differ from what is established to be the good adaptation to the IL of these activities [30]. When using games with competition, the teacher should seek a balance between award the team for winning the game or award a team/or an individual for cooperating in the game despite the final score. This is why it is important to bear in mind the type of game to be chosen according to the desired effects and the educational context of the intervention (see Figure 1).

#### 1.3.3. Phase 3: Actions during the Session or Learning Sequence

Once a PE session has begun, the teacher must follow the teaching strategies that he or she considers most appropriate to achieve the intended goals. In this model, it is important to encourage students’ awareness of their own MCs. The use of reflection-for, -in- and on-motor action will make it easier for each student to contextualize their interventions, in this case, concerning the motor interaction with the other participants and how possible interpersonal conflicts have been intervened [40,44,45,47].

Based on these considerations, the GIAM model proposes a series of actions aimed at one or more sessions (learning sequence) that the teacher can use during the development of her/his programming unit. These actions are based on the proposal of [64]:

(a) Action 1. Connector of previous knowledge. Moment of initial dialogue recalled, intending to intertwine previous knowledge with new learning. The teacher talks and reflects on what has happened in previous sessions, in this case, on the conflicting MCs; also asks and directs the different alternatives proposed by the students on how to intervene (reflection-on-action). This action does not need to be introduced in each session, it will depend on the conflicts observed in the previous session and how the learning sequence is progressing.
(b) Action 2. Building new knowledge. The teacher acts in the relationships between the class group and explains the activity (the rules of the game to be carried out). As for the relationships of the group after explaining the goals of the session [25,65], the teacher identifies the key elements, linking relational well-being with conflict transformation and expectations of respectful intervention during the game. Reflections arise around concepts such as empathy, respect, dialogue, effective communication, or effort, among others [9,36,37,38,40,66]. The intervention part in the class group does not need to be done in each session as it can be incorporated at any time of the learning sequence.

In both Action 1 and Action 2, the teacher can choose to create a space for strategic agreement, when collective games are used, between members of the same team. The reflection is aimed at preparing collective participation and considering the knowledge of relational well-being that should be respected considering previous experiences (reflection-for-action). This action favors the integration of new knowledge into the problem to be solved [67].

(c) Action 3. Holistic learning synthesis. The teacher tries to favor the development of the game, supervises the intervention of the players or teams and intervenes when in doubt about the rules of the game. She/he identifies adjusted motor and/or verbal conducts and misadjusted or perverse conducts that may generate or have originated some conflict. In the case of the presence of conflicts, she/he identifies its parts (action and reaction) and calibrates the magnitude of interpersonal tension (IC*f*). The teacher uses the didactic strategy of intervening in the game (modifying the rules to improve practice conditions and thus avoid the reproduction of new conflicts). When the teacher is forced to intervene in the protagonists of the conflict, he or she has different alternatives for them to make them reflect on their actions (reflection-in-action) [7,12,29,68,69,70,71,72]. Such action can become a key part of her/his educational process by acting directly on conflict transformation to improve the relational well-being of the class group. The proposals are as follows:
  - Momentary dialogue and “play on”. To separate the parties involved in the conflict with the intention that they find a solution to the problem encouraging reflection based on cooperative dialogue. It will not be necessary to take them away from the development of the game, being a minimal and momentary intervention.
  - Dialogue with momentary leave of the game. When the protagonists of the conflict cannot reach a rapid and consensual solution, it is convenient to temporarily remove them from the game. It is important to create a space for reflection based on cooperative dialogue without interrupting the progress of the task and altering the correct development of the class group. If the parties involved manage to find a solution to the problem, they will be able to return to practice.
  - Expulsion from the game. If a high-intensity conflict arises, the option of expulsion from the activity is chosen based on inappropriate behavior and against the requirements of the IL of the game. The members of the conflict are not withdrawn from the session by imposing a sanction [9], are only invited to abandon the task development, but remaining in the class. In the last part of the session, the teacher will use the reflection process based on cooperative dialogue (reflection-on-action) to re-engage students who have been withdrawn from the activity. The conflict situation with the parties involved in the conflict (internal parties) and the rest of the group (external parties) will be presented. The purpose of this action is to find a joint solution to the problem to be solved and to guarantee a quality learning process.

Accepting a respectful relationship with others means accepting the rules of the game. When this does not happen there is evidence to show that discomfort is generated in the participants due to the perception of the existence of a social injustice [43]. In this process, the teacher takes on the role of learning facilitator, mediator, and guide [9,37,39,40]. The aim is to encourage interpersonal dialogue among students in the search for alternatives to conflict. If the situation allows it, intervention on students in the first phases of the conflict will be avoided, favoring a space for reflection based on cooperative dialogue towards self-management and transformation of conflict situations [39,44,71,73]. Preventing students from developing a dependence on a third party to resolve future conflicts [43]. Becoming aware of and reflecting on one’s own MCs will help promote meaningful learning [74,75].

(d) Action 4. Reflection on the learning process. Reflective process based on a cooperative post-intervention dialogue (reflection-on-action). The teacher offers the students a reflective space of quality [76,77], emphasizing examples of adjusted individual and team MCs (that promote relational well-being) and samples of conflicting conducts that harm the climate of the class group. She/he encourages reflection on styles or attitude modes (competition, cooperation, evasion, submission and commitment) that she/he has observed facing possible conflicts. All this will contribute to the awareness of acquired learning; the one to be solved in real life [67] (see Figure 1).

After explaining the phases and actions of the GIAM model, this research aimed to determine the effect of the GIAM model on the relational well-being of third and fourth year ESO students in four secondary schools in Catalonia (Spain). The study hypothesized that the adjusted use of the GIAM model by PE teachers favors the improvement of their students’ relational well-being.

## 2. Method

### 2.1. Design

The study was an empirical research using an associative strategy to explore the relationship among variable, through a comparative study (exploration of four groups) [78]. The study was the result of a mixed-methods design [79,80,81], of triangulation of qualitative and quantitative data of the different instruments used. We performed the research in natural conditions with a phenomenological–interpretative perspective [82].

### 2.2. Participants

A total of 319 students (*M* = 14.89; *SD* = 0.65; *n_SS1_* = 82, *n_SS2_* = 91, *n_SS3_* = 46, *n_SS4_* = 100) from 3rd and 4th year of obligatory secondary school (ESO) participated in this study, 166 girls (52%) and 153 boys (48%). Nevertheless, a total of 32 students (10% of the sample) did not report data at both pre- and post-intervention and therefore were not included in subsequent analyses. Thus, all the analyses were conducted with a total sample of 287 valid students (*M* = 14.90; *SD* = 0.66). Specifically, an intentional probability sampling was carried out to choose secondary schools (SSs) which characteristics offered the presence of a diverse student body in the classrooms. Four SSs were selected in the provinces of Lleida and Tarragona (Spain) (see Table 2). The project was approved by the University of Lleida’s research ethics committee (UdL) (certificate with reference number 05/2019/CEICEGC). Furthermore, this project was presented and accepted by the territorial services of the Generalitat de Cataluña’s Department of Education (Spain) (delegations of Lleida and Terres de l’Ebre) and by the educational institutions involved in the research.

### 2.3. Instruments

Two instruments were used to analyze the degree of adjustment of the teacher’s intervention on the GIAM model and the effect on their students.

Teacher intervention (qualitative approach). The intervention of PE teachers and the participation of the researcher were considered. The role of the PE teacher was to lead the development of the session. Teachers’ interventions were recorded with an audio recorder. However, the researcher adopted the role of “participant observation”, from an emic viewpoint, using the field diary as a tool for collecting information on the development of the GIAM model [83]. In both cases, the transcriptions were made into a Microsoft Word document. Subsequently, the analysis of the content was carried out and the degree of adjustment of teachers to the GIAM model was identified. To this end, a dichotomous table was designed with the distribution of the actions and sub-actions of the teacher’s intervention, which determines all the important aspects to be controlled in the development of the model. Teacher interventions were considered to follow the GIAM model, provided that they were carried out at least 75% of the time in the course of the teaching intervention.

Effect on students (quantitative approach). We used the questionnaire “Peer Motivational Climate in Youth Sport Questionnaire” (PeerMCYSQ), validated into Spanish as “Clima Motivacional de los Iguales en el Deporte” by [84], to assess the orientation of the perceived motivational climate among peers in the same group (students of the four SSs) towards the task or towards the ego [85]. The questionnaire consists of 19 items preceded by the sentence, “In your class group, most of your classmates”, and grouped into three factors: task-climate (11 items; e.g., “the opinion of each individual is taken into account”); intra-team competition/ability (4 items; e.g., “they try to do better than their peers in the group “), and intra-team conflict (4 items; “they laugh at their colleagues when they make mistakes”). A 7-point Likert scale (1: strongly disagree and 7: strongly agree) is used to assess. The final score for each factor is calculated as an average of the items that are part of the scale.

### 2.4. Procedures

This study was part of a pedagogical experience designed to be applied in the ESO, with an educational and transformational purpose in search of the improvement of the relational well-being among the students in an educational community.

The teachers involved in the research received the information related to GIAM model about one month before the intervention. During this period all the questions and doubts were clarified in order to develop the GIAM model in optimal conditions. Consequently, this experience was determined to be performed at a certain time during the annual programming of the PE subject in each SS. The intervention on SS_1_ and SS_4_ was carried out in the last two months of the scholar course, whether the intervention at SS_2_ and SS_3_ was carried out in first two months of the following scholar course. For its evaluation, taking into account that it is an investigation involved in an educational process, a subjective attitudinal evaluation of the students about the development of the experience was chosen.

#### Pedagogical Intervention

An intervention (learning sequence) was designed based on the GIAM model. Seven sessions of 50 useful minutes each were held during four weeks aimed at educating the students’ relational well-being.

In the first session, an introduction to the model was made, explaining the development of the actions and strategies to be worked on during the sessions. Students were grouped into different heterogeneous groups following a stratified distribution of performance, remaining stable throughout the process (4 teams of 5–7 students in each group). Therefore, a first intervention was made with the Marro TSG to let the students know the functionality of the experience.

This was followed by the six remaining sessions following the format of an internal competition called “Marro League”. The students practiced four competitive sociomotor traditional sporting games of cooperation–opposition (TSGCOP): the marro, stealing stones, dodgeball, and pass the treasure (see Table 3) (see Table A1 in Appendix A).

The Marro game was the main protagonist of the process due to its high relational complexity and the lack of knowledge of it among the students [60]. Each TSGCOPs was structured in two phases: (a) a first phase called “strategic pact” (two minutes), where the students had a space for intra-team dialogue to propose a team strategy and agree on the knowledge of relational well-being to be respected in play (work on positive social skills), according to the social learning objectives and the climate to be generated in the session, depending on the requirements of the IL of the game; and (b) a second phase called “during the game” (seven minutes), where the different teams faced each other intending to obtain the maximum score in each match (inter-team).

Three rounds were held in each session; therefore, all the teams faced all their opponents. Within the competition’s framework, each team scored according to the result of the game (in each round). Nevertheless, it also provided a subjective score set by the teacher and the researcher (together), about the behavior and social interactions revealed by the group of students forming each team, with their peers (intra-team) and with their adversaries (inter-team). This score was evaluated taking into account key aspects of the theoretical framework of reference towards the transformation of conflicts through reflection-for, -in- and on-motor action: respect (for the rules of the game and others), self-esteem, empathy, cooperation, effective communication, self-regulation, emotional regulation and consensual decision-making.

The SSs teachers were responsible for applying the GIAM model and the learning sequence formed by TSGCOP. Therefore, they had to adapt their intervention to the demands of the model according to the different moments in which each session was structured.

### 2.5. Data Analysis

The data analysis is presented in two sections: (a) an analysis of the teachers’ intervention on the development of the experience and its level of adjustment to the characteristics of the GIAM model (qualitative analysis), and (b) analysis of the effects on the students’ perception of the motivational climate directed towards the task or the ego (intra-team competition/ability and intra-team conflict) (quantitative analysis).

On the one hand, for the analysis of qualitative data, a dichotomous instrument (see Table 4) was created to analyze the content of the recordings performed by the participating teachers in the study and of the researcher’s field diary about the degree of adjustment of teachers’ intervention to the GIAM model. To ensure the quality of the records, a comprehensive process involving 4 expert researchers (more than 10 years of experience in the field of conflict transformation in PE using the theory of motor action or motor praxeology) was carried out, following a process formed by 3 phases [86].

In a first phase, an ad hoc tool was designed based on the theoretical reference framework (theory of motor praxeology, reflective learning and conflictology), which brought together all the constituent actions and sub-actions of phase 3 of the GIAM model. In the second phase, an experts’ judgement was made. This procedure was performed twice, leaving a month between them [87]. Four researchers analyzed, individually, the content of the texts using the same categories and instrument. The successes and failures were then compared to ensure reliable matching using the Fleiss Kappa index (=0.81) allowing the quality of the records to be assured [88,89].

On the other hand, quantitative data was initially evaluated through preliminary analyses that included the study of data distribution. The descriptive statistics for all the variables in the study at pre- and post-intervention were also obtained. As a previous step before assessing the effects of the intervention, the measurement model of each instrument in the study was tested. All measurement models were estimated under the confirmatory factor analysis (CFA) approach using the Weighted Least Squares Means and Variance Adjusted (WLSMV) estimator in Mplus 7.0 software [90]. Model fit was assessed with the fit indices *χ*^2^ statistics, comparative fix index (CFI; [91]), Tucker–Lewis Index (TLI; [92]), and root mean square error of approximation (RMSEA; [93]) including its 90% confidence intervals (CI). According to [94], CFI and TLI values > 0.95 and RMSEA < 0.06 are considered as indicators of an excellent fit. In addition, CFI and TLI values > 0.90 and RMSEA < 0.08 are considered as indicators of acceptable fit [95].

To assess the effects of the intervention on the dependent variables, a repeated-measures ANOVA for each dependent variable was conducted, which is in line with previous studies that tested the effects of interventions in physical education classes (e.g., [96]). Dependent variables were students’ perceptions of task-climate, intra-team competition/ability and intra-team conflict. Three separate Group (SS_1_, SS_2_, SS_3_, SS_4_) X Time (Pre/Post intervention) repeated-measures ANOVAs were conducted. The statistic of interest was the attainment of a significant Group X Time interaction effect for each dependent variable—task-climate, intra-team competition/ability and intra-team conflict. Effect sizes were obtained via partial eta squared for each variable. In addition, we also carried out independent sample *t*-tests for each secondary school, to examine mean differences in the dependent variables from pre- and post-intervention. As a result of the multiple *t*-tests being performed during these analyses, we undertook a Bonferroni adjustment to the alpha level (new *p* = 0.004). ANOVA and *t*-test analysis was conducted using SPSS Statistics for Windows, version 17.0 (SPSS Inc., Chicago, IL, USA).

## 3. Results

### 3.1. Analysis of the Adaptation of Teachers’ Intervention on the GIAM Model

The content analysis shows differences in the degree of adjustment of teacher intervention on the GIAM model by teachers of four SSs. The teachers corresponding to SS_1_ and SS_2_ adapted their intervention to all those actions and sub-actions of the GIAM model during the development of the pedagogical intervention (20 items 100%). In contrast, only 50% of the items for SS_3_ (10 items 50%) and 35% for SS_4_ (7 items 35%) (see Table 4). Considering the items listed in Table 4 about the actions and sub-actions of the development of the GIAM model:

(a) In the four SSs, the learning outcomes and rules of the TSG used during this pedagogical experience were explained, allowing students to ask questions and appropriate time (reflection-for-action) was also provided to agree on team strategy before the game (AS/SLS_2_). In all SSs the correct functioning of the game and equipment was monitored, intervening when students ask for help or to clarify some rules of the game. All the SSs intervened on the game’s IL given the origin of conflicts arising from the conditions of the game (AS/SLS_3_).

(b) The teachers from SS_3_ and SS_4_ did not encourage the process of reflection on the experience of conflict situations in previous sessions (reflection-on-action). Neither did they direct the proposal of student alternatives towards a win-win attitude mode for the management of future similar situations (AS/SLS_1_). They did not clearly explain what aspects should be considered when transforming conflicts into a proper interpersonal relationship with colleagues in the group (empathy, respect, active listening, etc.). Additionally, they did not pay attention to the rules’ agreement reached previously, right before the development of the game (reflection-for-action) during the AS/SLS_2_. These two teachers did not use an indirect style, they did not offer the students time to self-manage and transform their conflicts, nor did they use reflection on the intervention about the protagonists of the conflict (reflection-in-action) during the AS/SLS_3_.

(c) In the SS_3_ the presence of conflicts between students during the game was identified (AS/SLS_3_), unlike in SS_4_.

(d) In the SS_3_ there was a reflection on the interpersonal relationship between partners and adversaries, emphasizing some of the MCs and/or VCs adjusted during the game; but the misadjusted and perverse MCs and/or VCs linked to the IL of the game were not addressed nor the attitude mode of conflict management during AS/SLS_4_. In the SS_4_ no sub-action was carried out of those covered by the reflection process during the AS/SLS_4_.

### 3.2. Preliminary Analyses about the Students’ Perception

Table 5 presents the mean and standard deviation for each dependent variable at each time point (i.e., pre- and post-intervention) and for each SS. Overall, values of asymmetry and kurtosis supported the use of parametric tests. Regarding the confirmatory factor analysis, fit-indices show that PeerMCSYQ measurement models had an acceptable fit to the data: pre-intervention, *χ*^2^ (*df*) = 385.891 (149), *p* < 0.001, RMSEA (90% CI) = 0.072 (0.063–0.080), CFI = 0.941, TLI = 0.932; post-intervention, *χ*^2^ (*df*) = 343.374 (149), *p* < 0.001, RMSEA (90% CI) = 0.066 (0.057–0.076), CFI = 0.959, TLI = 0.953.

### 3.3. Change in Dependent Variables from Pre- to Post-Intervention about the Students’ Perception

In this subsection, we examine whether the effects of the intervention on students’ perceptions of task-climate, intra-team competition/ability, and intra-team conflict would be different depending on their SSs. Table 6 presents the results of the repeated-measures ANOVA. As can be observed, there was an interaction effect between the secondary school and the intervention for the students’ perceptions of task-climate (*p* = 0.020). This indicates that the intervention had different small effects on the students’ perceptions of task-climate depending on their SS (*η*^2^ = 0.034). There were no interaction effects for neither intra-team competition/ability (*p* = 0.191) nor intra-team conflict (*p* = 0.105).

If we consider the results of all the groups in general, we observe that there were significant differences in the effect of the intervention (i.e., GIAM model). Students increased their scores for task-climate (*p* < 0.001, *η*^2^ = 0.195) and decreased their scores for intra-team competition/ability (*p* = 0.009, *η*^2^ = 0.024) and intra-team conflict (*p* < 0.001, *η*^2^ = 0.144) from pre- to post-intervention.

Paired-sample *t*-tests (see Table 7) for each SS revealed significant differences in the pre-post comparison involving the dependent variables. Regarding the students’ perceptions of task-climate, all SSs but SS_3_ significantly increased their scores from pre- to post-intervention. In addition, no differences were observed in intra-team competition/ability in none of the secondary schools (*p* > 0.004). Finally, concerning intra-team conflict, all SSs but SS_3_ significantly decreased their scores from pre- to post-intervention.

## 4. Discussion

This research aimed to determine the effect of the GIAM model on the relational well-being of third and fourth year ESO students in four schools in Catalonia (Spain). The correct execution of the GIAM model by PE teachers was the starting hypothesis proposed to demonstrate the improvement of the relational well-being of their students.

The data illustrate that after the pedagogical intervention, significant changes were generated in favor of relational well-being. Student perception scores increased in the task-climate and decreased in the intra-team conflict factor. These results show that the application of the GIAM model contributed to generate a teaching–learning (T–L) context based on the creation of a positive motivational climate [8,11,13,14].

To this end, it should be noted that these results were obtained by an educational experience based on competitive TSGCOP, whose IL is associated with a team duel. That is to say, the games proposed have an internal logic whose system of relationships corresponds to the win–lose model (one team wins and the other loses) [25,42,57]. It is an educational experience in which the participation of the students in this team duel makes possible that the possible conflictive relationships with the others are oriented towards a win–win model of coexistence [30,35,41]. Students need to be able to see all other participants (partners and adversaries) as allies in that collective experience. When they learn to recognize the other players as allies, then the teacher could influence the positive motivational climate of the students. She/he should highlight that the process peaceful relationships are more important than just the result (winning or losing). Intervening on the motivational climate in physical education classes can change the number of potentially conflicting situations [13,19,30].

A great educational challenge for students who should learn to recognize any conflict as an opportunity for democratic coexistence and mutual respect [7,10,29,30].

### GIAM Model: A Pedagogical Model towards a Win–Win Style or Attitude Mode

All SSs developed the GIAM model based on two preparatory phases (phase 1 and 2) prescribed by the researchers. After taking part in those phases, the study determined the extent to which teachers followed the actions and sub-actions of phase 3. The results reflect the fact that they are mostly in line with action 2; however, they had difficulties in following the guidelines of the GIAM model in actions 1, 3 and 4.

Furthermore, the results show constant attention of the teachers on the correct functioning of the game or learning sequence, but on the other hand, they present greater difficulties when intervening in the presence of conflicts (action 3).

Let us remember that according to the theoretical framework of reference, the conflict in the game has two parts, one origin (caused by an action of play: VCAS, MMC, PMC, (misadjusted or perverse)) and a reaction (verbal, physical, or mixed aggression) [26,28,31]. Various studies recommend acting on these two phases according to the characteristics of the conflict and its degree of intensity (ICf) [29,30]. The four teachers modify the rules of the game in an appropriate way when the origin is related to the conditions established by themselves. However, only two teachers (SS_3_ and SS_4_) do not know how to intervene on the protagonists of the conflict to favor the regulation of negative emotions and guide the self-management of the conflict in search of an attitude mode based on the win–win.

First of all, students need to know what elements should include a proper relationship with their colleagues and adversaries. Working on empathy, active listening, autonomy, cooperation, among others, are key elements for positive conflict transformation [9,38,39,40]. During action 2, two teachers (SS_3_ and SS_4_) did not facilitate the initial dialogue to raise awareness of these issues and offer resources to their students.

Secondly, to move towards a collaborative attitude, the teaching strategy based on reflection becomes another fundamental tool for placing students as the protagonists of the T–L process [40,49,50,52]. Using a reflective process for-in-on motor action allows students to become aware of how they have related to others [44]. Reflection helps students to identify the behaviors involved, their level of adaptation to peaceful coexistence and the attitude they have used in the face of possible conflicts [44,45,47].

The SS_3_ and SS_4_ teachers did not offer students the possibility of participating in an initial process of reflection and dialogue, which was always recalled. When they intervened on the protagonists of the conflict, they did not use reflection based on a constructive and cooperative dialogue. Nor did they re-expose the conflictive behaviors present during the game in a final group reflection process.

The results of the students’ perception of the motivational climate reinforce the evidence presented so far. All the SSs in which the experience took place did not behave in the same way. In the SSs where the proposed model was followed (SS_1_ and SS_2_), a similar T–L context was offered to their students. In these two centers a greater positive motivational climate was generated, oriented towards the task-climate and the presence of the intra-team conflict factor was reduced (ego-orientation); aspects related to the improvement of relational well-being [2,14,18,19,40].

On the other hand, it is interesting to note the uneven effect of the GIAM model upon the motivational climate of students in SSs where the model was not fully followed (SS_3_ and SS_4_). Depending on the adaptation and correct execution of the actions, sub-actions and didactic strategies corresponding to phase 3 of the GIAM model, the orientation of the motivational climate towards the task-climate can have a greater or lesser effect on the participants. Surprisingly, the SS_4_, that did not adapt to the GIAM model, also leads to significant changes in favor of relational well-being. Because of the complexity of this issue, this study considers the need for further research to determine what other elements may be involved in addition to the teacher’s intervention. According to Sáez de Ocáriz and Lavega-Burgués [30], the importance of having instruments available to know the students’ perception of the experience of the conflict and the phases that constitute it (action and reaction) is reinforced.

As limitations of the study, it should be noted that the GIAM model has only been applied to four SSs during the development of a seven-session learning sequence. As a future perspective, it would be interesting to contemplate the application of the model in a greater number of SSs, as well as to see the effectiveness of the model applied in different long-term learning sequences (e.g., full academic year).

## 5. Conclusions

This research provides new scientific evidence in the field of PE to take another step forward to improve relational well-being and school coexistence.

Conflict is a phenomenon that is part of the past, present and future of educational institutions and, of course, part of anyone’s life. The results show that the GIAM model, as a pedagogical model [55], can be a useful tool at the disposal of teachers for conflict transformation. It has been seen as a valid model for educating relational well-being and responding to one of the great challenges facing teachers in the 21st century, learning to live together in society [1,4,9]. This model offers the teacher or monitor the opportunity to transform the attitude of students towards situations of empathy, cooperation, active listening, and consensual decision-making, which is to say, towards a collaborative attitude in which the protagonists of the conflict can win (win–win attitude mode).

A key aspect of this model is that the teacher is able to identify the conflicts in the game, recognize their parts and their rate of conflict. She/he must then decide whether to intervene on the modification of the rules of the game or on the participants, going through processes based on reflection-for, -in- and on-motor action.

Motor Praxeology, Reflective Learning and Conflictology, are the pillars that support the development of the GIAM model under a vision of a modern, reflective, peaceful, affective, and relational PE. Considering these characteristics, it is a multipurpose model to be used in different contents.

## Figures and Tables

**Table 1:** Styles or attitude modes of conflict management associated with winning the game (adapted from [35]).

Ways of Dealing with Conflict	Importance of Dimensions	Winning in the Game
Collaborating	High assertiveness—High cooperativeness	Win–Win
Competing	High assertiveness—Low cooperativeness	Win–Lose
Accommodating	Low assertiveness—High cooperativeness	Lose–Win
Avoiding	Low assertiveness—Low cooperativeness	Lose–Lose
Compromising	Medium assertiveness—Medium cooperativeness	Negotiation

**Table 2:** Mean and standard deviation of the study sample.

SS	*n*	Course	Boys				Girls			
			*n*	%	*M*	*SD*	*n*	%	*M*	*SD*
SS_1_	75	3º	32	42.7%	14.50	0.51	43	57.3%	14.72	0.45
SS_2_	81	4º	37	45.7%	15.51	0.51	44	54.3%	15.66	0.48
SS_3_	45	3º	26	57.8%	14.73	0.45	19	42.2%	14.47	0.61
SS_4_	86	3º	46	53.5%	14.67	0.47	40	46.5%	14.60	0.50

Note: SS = Secondary School; Course = academic course (ESO); *M* = Mean; *SD* = Standard deviation.

**Table 3:** Learning sequence schedule. Distribution of competitive TSGCOPs in each session.

Session	Competitive Traditional Sporting Games of Cooperation-Opposition
1	Introduction and Marro
2	Marro
3	Stealing stones
4	Marro
5	Dodgeball
6	Pass the treasure
7	Marro

**Table 4:** Actions and sub-actions of the teaching intervention according to the GIAM model.

Actions and Sub-Actions of the Teaching Intervention		Secondary Schools				
		SS_1_	SS_2_	SS_3_	SS_4_	SS_T_
AS/SLS 1. Connector of previous knowledge						
Initial recalled dialogue	Reflects on what has happened in previous sessions	✓	✓	✕	✕	2
	Leads the dialogue into the different alternatives proposed by the students towards a way to face the collaborating conflict (win–win)	✓	✓	✕	✕	2
AS/SLS 2. Building new knowledge						
Group system relations	Explains learning outcomes in the interpersonal relationships	✓	✓	✓	✓	4
	Clearly explains what an appropriate relationship with other participants should include	✓	✓	✕	✕	2
	Links the way of relating to others to conflict transformation	✓	✓	✕	✕	2
	Offers the possibility to ask	✓	✓	✓	✓	4
Traditional Sporting Game	Explains the rules of the game	✓	✓	✓	✓	4
Intra-Team Strategic Pact	Establishes an appropriate time when she/he offers the possibility of agreeing on equipment	✓	✓	✓	✓	4
	Pays attention to the teams who agree and intervenes if necessary	✓	✓	✕	✕	2
AS/SLS 3. Holistic learning synthesis						
Teacher’s intervention for the correct development of the game	Supervises the development of the game and the correct functioning of the teams	✓	✓	✓	✓	4
	Indirectly supports teams/students when necessary	✓	✓	✕	✕	2
	Intervenes at the request of help or if she/he needs to clarify the rules of the game	✓	✓	✓	✓	4
Teacher’s intervention in the presence of conflicts in the game	Identifies the presence of conflicts during the game	✓	✓	✓	✕	3
	Gives priority to the initial intervention of students in the presence of conflicts	✓	✓	✕	✕	2
	Intervenes by modifying the rules of the game	✓	✓	✓	✓	4
	Uses reflection-in-motor action to optimize win–win attitude mode	✓	✓	✕	✕	2
AS/SLS 4. Reflection on the learning process						
Reflective process	Reflects on the type of interpersonal relationships between partners and opponents during the game	✓	✓	✓	✕	3
	Highlights adjusted MCs and/or VCs in interpersonal relationships between allies and opponents	✓	✓	✓	✕	3
	Exposes conflicting interpersonal relationships linked to the rules of the game or the attitude mode adopted by the students	✓	✓	✕	✕	2
	Invites to favor a win–win attitude mode towards conflicting interpersonal relationships in future sessions	✓	✓	✕	✕	2
Summary (20 items)		20	20	10	7	
*%*		100%	100%	50%	35%	

Note: SS = Secondary School; AS/SLS = Actions during the session or sessions of a learning sequence; SS_T_ = adjustment to the item by the four SS.

**Table 5:** Descriptive of the pre- and post-intervention dependent variables for each SS.

SS	Variables	Pre-Intervention				Post-Intervention			
		*M*	*SD*	Skew	Kurt	*M*	*SD*	Skew	Kurt
SS_1_ *n* = 75	Task-climate	4.03	0.90	0.07	1.70	4.63	1.17	−0.38	−0.07
	Intra-team competition/ability	5.25	0.96	−0.34	0.03	4.89	1.14	−0.42	−0.57
	Intra-team conflict	4.49	1.13	−0.47	−0.10	3.74	1.34	0.12	−0.57
SS_2_ *n* = 81	Task-climate	3.97	1.04	−0.17	−0.18	4.92	1.27	−0.80	0.00
	Intra-team competition/ability	4.94	1.11	−0.42	−0.54	4.50	1.22	−0.33	−0.24
	Intra-team conflict	4.35	1.39	−0.01	−0.67	3.34	1.56	0.60	−0.09
SS_3_ *n* = 45	Task-climate	5.19	0.91	−0.22	−0.73	5.56	1.08	−0.96	0.69
	Intra-team competition/ability	4.83	1.04	0.10	−0.74	4.83	1.20	−0.38	−0.17
	Intra-team conflict	2.86	1.40	0.75	−0.35	2.44	1.40	1.58	2.80
SS_4_ *n* = 86	Task-climate	4.42	1.16	−0.20	−0.38	4.88	0.86	−0.49	0.01
	Intra-team competition/ability	5.18	1.09	−1.30	2.43	5.10	0.95	−0.23	−0.27
	Intra-team conflict	4.10	1.43	−0.29	−0.62	3.61	1.31	0.01	−0.64

Note: SS = Secondary School; *M* = Mean; *SD* = Standard deviation; Skew = Skewness; Kurt = Kurtosis. All scales were responded using a 7-point Likert scale.

**Table 6:** ANOVAs on the different variables.

Variables	Time			Secondary School			Time X Secondary School		
	*F*	*p*	*η* ^2^	*F*	*p*	*η* ^2^	*F*	*p*	*η*^2^
Task-climate	68.54	<0.001	0.195	14.59	<0.001	0.134	3.34	0.020	0.034
Intra-team competition/ability	6.91	0.009	0.024	4.37	0.005	0.044	1.60	0.191	0.017
Intra-team conflict	47.72	<0.001	0.144	17.29	<0.001	0.155	2.06	0.105	0.021

Note: *F =* result of the F test; *p* = significance, *p* < 0.05; *η*^2^ Partial = partial eta-squared effect size.

**Table 7:** Mean difference between pre- to post-intervention scores.

	Task-Climate			Intra-Team Competition/Ability			Intra-Team Conflict		
SS	*M diff.*	*t*	*p*	*M diff.*	*t*	*p*	*M diff.*	*t*	*p*
SS_1_	0.60	4.33	<0.001	−0.36	−2.19	0.032	−0.75	−4.16	<0.001
SS_2_	0.95	7.23	<0.001	−0.44	−2.88	0.005	−1.01	−5.57	<0.001
SS_3_	0.37	2.19	0.034	0.00	0.00	1.000	−0.42	−1.62	0.113
SS_4_	0.46	3.63	<0.001	−0.08	0.57	0.572	−0.49	−3.09	0.003

Note: SS = Secondary School; *M diff.* = Mean difference between pre- and post-intervention scores. Bonferroni adjustment to the alpha level was *p* = 0.004.

## Appendix A

**Table A1:** Description of the competitive TSGCOP used in the study.

Description of Competitive Sociomotor Traditional Sporting Games of Cooperation–Opposition
Marro
Team duel, with uniform space and no use of material. Two teams of the same number of players face each other intending to catch as many opponents as possible before the end of the game. At the beginning, a player will leave her/his house (baseline) and say, “Marro!”; if an opponent says, “Marro!” and comes out later, she/he can catch the first player. Whenever a participant enters the playing field, she/he will be vulnerable to all the opponents who will leave the playing field after her/him. At any time, a player can return to her/his home to go out again when she/he feels it is necessary. Any participant who is in prison can return to the field if a member of her/his team touches her/his hand.
Stealing stones
Team duel, with uniform space and use of material: cones (stones). Two teams of the same number of players face each other intending to get as many stones as possible before the end of the game. Each team has 20 stones in their field. The teams are structured in two defensive players (only they can catch the opponents when they cross the midfield line and take them to the prison) and the rest, attackers (only they can go to the opposite field and try to steal stones). Any participant who is in prison can return to the field if a member of her/his team touches her/his hand.
Dodgeball
Team duel, with uniform space and use of material: ball. Two teams of the same number of players face off with the aim of “killing” (an opponent is hit by the ball and it touches the ground) all players of the opposing team. When a player is hit by the ball, should be directed to the cemetery (the baseline of the opposing team) to throw the ball and try to touch a participant from the opposing team. If she/he succeed, she/he can return to the field with her/his team. If a player who has been thrown the ball can hold it before it touches the ground, she/he is not eliminated.
Pass the treasure
Team duel, with stable/uniform space and use of material: small object (treasure). Two teams of the same number of players face each other intending to bring the treasure to the rival team’s baseline. The two teams leave from their camp, one of them with an attacking role (they carry the treasure), the others with the advocacy role (they must find the treasure). Only one player of the attacking team carries the treasure in his hand. When a defender touches an attacker, the attacker finds out if he is the bearer of the treasure, but both are out of play.
